# Editorial: The cerebellar role in psychiatric disorders: Emerging evidence and future perspectives

**DOI:** 10.3389/fnbeh.2022.1075679

**Published:** 2022-11-01

**Authors:** Georgios P. D. Argyropoulos, Michela Lupo, Giusy Olivito

**Affiliations:** ^1^Division of Psychology, Faculty of Natural Sciences, University of Stirling, Stirling, United Kingdom; ^2^Servizio di Tutela della Salute Mentale e Riabilitazione dell'Età Evolutiva Azienda Sanitaria Locale, Rome, Italy; ^3^Department of Psychology, Sapienza University of Rome, Rome, Italy; ^4^Ataxia Laboratory, Istituto di Ricovero e Cura a Carattere Scientifico Fondazione Santa Lucia, Rome, Italy

**Keywords:** cerebellum, emotion, affect, social cognition, Theory of Mind

Over the past decades, clinical, neuroimaging, anatomical, and physiological studies have established the presence of a “cognitive” and a “limbic” cerebellum—the former being represented primarily in posterolateral regions and the dentate nuclei, and the latter in the vermis and the fastigial nuclei (Schmahmann et al., 2007). The “dysmetria of thought,” following damage to the cognitive cerebellum (Schmahmann, 1998) and the neuropsychiatric impairments, following damage to the limbic cerebellum (Schmahmann et al., 2007) comprise the so called “cerebellar cognitive affective syndrome” (Schmahmann and Sherman, 1998). These findings have recently renewed interest in a cerebellar pathophysiology of a broad range of neurodevelopmental and psychiatric disorders (e.g. Hoppenbrouwers et al., 2008; Lupo et al., 2019; Van Overwalle et al., 2020).

Nevertheless, the field remains in its infancy, especially when considering the markedly different symptoms of these disorders.

The goal of this Research Topic is thus to contribute toward: (i) identifying structural/functional properties of the cerebellum and its networks in conditions that impact emotion, affect, and social cognition; (ii) designing therapeutic interventions and assessing their efficacy on the basis of theories of cerebellar function. The Research Topic comprises four original articles, focused on high-level social sequencing in Autism Spectrum Disorders (ASD; Bylemans et al.; Heleven et al.), Theory of Mind (ToM) deficits in Bipolar Disorder (BD) types 1 and 2 (BD1, BD2; Olivito et al.), and trait-depression and hyperactivity (Jackson and Bernard), all in relation to theories of cerebellar function or structural/functional properties of the cerebellum and its networks.

The papers by Bylemans et al. and Heleven et al. report work in adults with ASD based on the cerebellar “sequence detection” hypothesis (Leggio and Molinari, 2015). Within this framework, the core, multi-domain operation of the cerebellum is the detection, automatization, simulation, and correction of sequences. Building on work that implicates this cerebellar function in social mentalising (Van Overwalle et al., 2014, 2019; Heleven et al., 2019) and studies associating cerebellar abnormalities with several aspects of ASD (e.g., D'Mello et al., 2015; Olivito et al., 2018), Heleven et al. hypothesized that social sequence processing is impaired in ASD. They thus compared high-functioning individuals with ASD with neurotypical controls on the Picture and Verbal Sequencing Tasks (Heleven et al., 2019). In these tasks, participants place events (mechanical events devoid of a social aspect; social routines; high-level social beliefs) in the correct chronological order. While accuracy rates were at ceiling levels for both groups and both tasks, the Picture Sequencing Task showed longer response latencies for individuals with ASD relative to controls for events involving social routines/beliefs, but not for non-social mechanical events. This study is one of the few to investigate high-level social sequencing in individuals with ASD, and highlights the utility of this task in behavioral-clinical research.

Within the same framework, Bylemans et al. developed a narrative sequencing and mentalizing training program for adults with ASD. Its novelty is highlighted by the fact that research has been predominantly focused on children, and that the few programs available for adults remain devoid of concrete neuroscientific foundations. The authors randomly assigned participants with ASD to either a Training group or a waiting-list Control group. The former underwent sessions where participants narrated stories adopting the perspective of the original storyteller and answered questions requiring mentalizing. Relative to the Control group, the Training group significantly improved in mentalizing about others' beliefs and in narrative coherence immediately post-training, relative to pre-training. Moreover, almost all members of that group expressed beneficial effects on their mood, with half of them reporting positive effects on their self-confidence in social situations. These results highlight the utility of neuroscience-informed therapeutic interventions for ASD. Given the evidence for cerebellar involvement in sequencing social actions and metalizing, the authors reason that their training program in the future could be augmented by non-invasive cerebellar stimulation.

Olivito et al. present the first study to differentiate BD1 and BD2 on ToM deficits and cerebellar abnormalities. While the latter have been previously reported (e.g., Lupo et al., 2021; Olivito et al., 2022), their relationship with ToM deficits (e.g., Samamé et al., 2012) remains underexplored. The authors compared BD1 and BD2 patients in the euthymic phase with healthy controls on tests evaluating different aspects of ToM. BD1 patients showed deficits in cognitive and advanced components of ToM, while BD2 patients showed more pronounced deficits, extending to affective and automatic components. The authors also compared cerebellar gray matter volumes of BD1 and BD2 patients with those of controls. The regions of volume reduction only partially overlapped in the two BD groups (primarily in Crus II). Importantly, in both BD1 and BD2, ToM scores positively correlated with the reduced cerebellar volumes, suggesting that differences in structural abnormalities may account for those in ToM deficits between BD1 and BD2.

Functional network properties also help illustrate the neurobiological foundations of psychiatric disorders: Jackson and Bernard focused on resting-state functional cerebellar-basal ganglia networks. Their approach was informed by evidence for: (i) both direct and indirect anatomical connectivity between the cerebellum and the basal ganglia in non-human primates (cf. Bostan and Strick, 2018); (ii) the involvement of both structures in the pathophysiology of several neuropsychiatric/neurodevelopmental disorders (e.g., Hoppenbrouwers et al., 2008; Macpherson and Hikida, 2019); (iii) a distinction between cognitive and motor cerebellar-basal ganglia networks (Jackson and Bernard, 2022). Here, the authors addressed the question whether integration in cognitive or motor cerebellar-basal ganglia networks predicted self-reported psychiatric measures and cognitive task performance in a large community sample of young adults. Their results showed that, while cognitive network integration marginally predicted a positive relationship with hyperactivity, motor network integration negatively predicted hyperactivity and depression.

Overall, this Research Topic highlights (i) the importance of structural/functional abnormalities of the cerebellum and its networks in the neurobiology of disorders that compromise emotion, affect, and social cognition; (ii) the significance of incorporating theories of cerebellar function in studying these disorders and in developing therapeutic programs; (iii) the potential of using cerebellar sites as targets for neuromodulation within the context of adjuvant therapeutic interventions.

## Author contributions

GA compiled the first draft of the manuscript. GO and ML reviewed and finalized the manuscript. All authors have read and approved the final version of the article for publication.

## Conflict of interest

The authors declare that the research was conducted in the absence of any commercial or financial relationships that could be construed as a potential conflict of interest.

## Publisher's note

All claims expressed in this article are solely those of the authors and do not necessarily represent those of their affiliated organizations, or those of the publisher, the editors and the reviewers. Any product that may be evaluated in this article, or claim that may be made by its manufacturer, is not guaranteed or endorsed by the publisher.
